# Minimum Length Scheduling for Multi-Cell Full Duplex Wireless Powered Communication Networks

**DOI:** 10.3390/s21196599

**Published:** 2021-10-02

**Authors:** Muhammad Shahid Iqbal, Yalcin Sadi, Sinem Coleri

**Affiliations:** 1 Department of Electrical Engineering, National University of Technology, Islamabad 44000, Pakistan; 2 Department of Electrical and Electronics Engineering, Kadir Has University, Istanbul 34083, Turkey; yalcin.sadi@khas.edu.tr; 3 Department of Electrical and Electronics Engineering, Koc University, Istanbul 34450, Turkey; scoleri@ku.edu.tr

**Keywords:** wireless powered communication networks, energy harvesting, full-duplex, power control, scheduling, multi-cell network

## Abstract

Wireless powered communication networks (WPCNs) will be a major enabler of massive machine type communications (MTCs), which is a major service domain for 5G and beyond systems. These MTC networks will be deployed by using low-power transceivers and a very limited set of transmission configurations. We investigate a novel minimum length scheduling problem for multi-cell full-duplex wireless powered communication networks to determine the optimal power control and scheduling for constant rate transmission model. The formulated optimization problem is combinatorial in nature and, thus, difficult to solve for the global optimum. As a solution strategy, first, we decompose the problem into the power control problem (PCP) and scheduling problem. For the PCP, we propose the optimal polynomial time algorithm based on the evaluation of Perron–Frobenius conditions. For the scheduling problem, we propose a heuristic algorithm that aims to maximize the number of concurrently transmitting users by maximizing the allowable interference on each user without violating the signal-to-noise-ratio (SNR) requirements. Through extensive simulations, we demonstrate a 50% reduction in the schedule length by using the proposed algorithm in comparison to unscheduled concurrent transmissions.

## 1. Introduction

According to the recent Ericsson mobility report, 24.6 billion sensor nodes are expected to be installed by 2025 [1]. The majority of the applications in real time monitoring and industrial automation have strict delay requirements [2]. Therefore, increasing the lifetime of this massive number of sensor nodes while satisfying their latency requirements is a major challenge for future wireless networks. Moreover, to fulfill these diverse requirements, this massive installation will be deployed by using low power transceivers with energy harvesting capability, simple receiver circuitry, and intelligent medium access protocols [3].

For perpetual energy, due to full control of the energy transfer, small form factor, long range and no alignment requirements, the radio frequency (RF) energy harvesting (EH) is a promising technique for the wireless energy transfer for IoT and MTC type applications. In the literature, RF-EH has been investigated with two models named the simultaneous wireless information and power transfer (SWIPT) and wireless powered communication network (WPCN). In SWIPT, the same signal is used for simultaneous energy and information transfer.

The authors in [4,5] analyzed the trade off between energy efficiency and information transmission capacity for a single user using additive white Gaussian noise and a flat fading channel, respectively. The multi-user SWIPT was investigated for data buffer constrained sum throughput maximization [6] and energy efficiency maximization under the energy causality constraint [7].

On the other hand, WPCN consists of a hybrid access point (HAP), which is responsible for dedicated energy transmission in the downlink and a set of users that harvest this energy to transmit back their information to the HAP in the uplink. The authors in [8] introduced the first protocol for the sum throughput maximization of a WPCN, in which the frame length is divided into two non-overlapping phases dedicated for energy and information transfer. In this half-duplex model, as energy and information take place in different phases, the information transmission order of the users is not important, thus, no scheduling algorithm is required.

To increase the energy harvesting rate of such networks, the usage of multiple energy transmitters is investigated in [9,10,11]. Particularly, [9] studied the time allocation and load balancing for the throughput maximization, [10] provided the implementation of a test-bed to maximize the amount of harvested energy, and [11] investigated a wireless body area network with the objective of maximizing the sum throughput. Furthermore, to increase the network coverage, multi-cell WPCN, in which multiple HAPs are used for both energy transmission and information reception, were investigated for minimum throughput maximization [12] and throughput maximization through beam-forming [13].

However, these objectives fail to provide any delay guarantee, which is necessary for the time critical networks. Only [14] considered the minimum length scheduling problem. All of these problems investigated for multi-cell network consider half-duplex model, which results in an under utilization of the time and energy resources due to non-overlapping energy and information transmission phases.

### 1.1. Related Works

Due to the recent advances in self-interference cancellation techniques for wireless networks [15,16,17,18] and the related practical implementations [19,20], full-duplex has become a potential transceiving technique for 5G and beyond networks. In full-duplex, the uplink and downlink transmissions take place simultaneously using the same frequency reducing the spectrum requirements to half, i.e., either the spectral efficiency or the total number of users within a cell zone can be increased by a factor of two. From a practical implementation point of view, [21] presented an experimental setup for low complexity full-duplex design by employing the passive suppression with a patch antenna and active self-interference cancellation by using estimation and reconstruction of the interfering signal.

The authors in [22] implemented a prototype for full-duplex operation by implementing a wide-band RF canceller and an adaptive non-linear digital canceller. In the context of full-duplex networks, [23] categorized all the users into lethargic users, i.e., users with low energy levels and energetic users, i.e., the users with sufficient energy stored in their batteries. The authors proposed a medium access protocol named FarMac, which aims to maximize the energy transferred to the lethargic users and information received from the energetic users.

The authors in [24] investigated the minimum length scheduling problem for a single cell continuous rate transmission model. To solve the problem, the authors used a penalty function defined as the difference between the actual transmission time and the minimum transmission time of a user at a particular scheduling decision time. Since the penalty function is purely characterized for a single user, the solutions are not applicable for concurrent transmissions. The authors in [25] solved the sum throughput maximization problem for a single cell continuous rate model.

The continuous rate model is hard to realize practically for such low energy harvesting networks. To address this issue and to simplify the receiver structure, The authors in [26,27,28] studied an on–off transmission scheme for a single cell network with the objective to derive the outage probability, minimize the schedule length, and maximize the sum throughput, respectively. In an on–off transmission scheme, the users either transmit information using the maximum allowed transmit power or remain silent if they do not not have enough power to achieve this power level throughout their transmission.

The researchers in [3,29,30] further extended these formulations to investigate the minimum length scheduling problem and sum throughput maximization problem for a discrete rate model, where any finite set of discrete transmission rate values are supported. In [3], the authors defined the minimum length scheduling (MLS) slot, which is defined as the slot corresponding to the minimum transmission completion time while starting anytime after the scheduling decision time.

The scheduling problem is solved based on the allocation of the MLS slots to the users. The MLS slot is purely defined for a single user without considering the concurrent transmissions and inter-cell interference. Since the MLS slot specifically focuses on the first time instant at which transmission is feasible for a specific user; it is not generalizable to multi-cell networks in which the feasibility of the transmission of a particular user depends on the other concurrently transmitting users. All of these studies consider a single cell network, which limits their wide deployment due to low energy harvesting rate, limited applications and impractical model for wide coverage, which does not consider the inter-cell interference.

The major challenge for multi-cell WPCN is to determine the group of users that will transmit simultaneously. For a single cell scenario, it is easy to come up with some certain characteristics that can help to determine the optimal solutions. However, for a multi-cell scenario, the per user characteristic based solutions, such as penalty or MLS slot-based solutions, are not applicable since the transmit powers, transmission rates, interference, and energy harvesting characteristics are now dependent on a set of concurrently transmitting users rather than the characteristics of a single user.

In [3,24], the authors proposed solutions relying on the characteristics of the user without considering the other users; however, in the multi-cell scenario, we need to consider all the simultaneously transmitting users. To the best of the authors’ knowledge, this is the first study to investigate the multi-cell full-duplex WPCN while considering the self-interference and inter-cell interference for a simultaneous transmission of the users.

### 1.2. Contributions

The goal of this paper is to determine the optimal power control and scheduling for the constant transmission rate model with the objective of minimizing the schedule length for a realistic multi-cell full-duplex WPCN system. In full-duplex, all the users can harvest energy during the information transmission of other users. Therefore, the users with lower energy level can be scheduled later so that they harvest more energy and can transmit their information in shorter time. Moreover, in multi-cell WPCN, the users transmitting information simultaneously interfere with each other, which necessitates an intelligent grouping mechanism to minimize the interference among the concurrently transmitting users. The original contributions of this paper are listed below:

- We propose an optimization framework for the minimization of the schedule length in a full-duplex multi-cell WPCN for the first time in the literature. The framework considers the concurrent transmissions of the users, and incorporates the non-linear energy harvesting model for a full-duplex WPCN, in which the HAPs and users both operate in full-duplex mode.
- We formulate a mixed-integer non-linear optimization problem to minimize the schedule length. The formulated optimization problem is non-convex and generally hard to solve for the global optimal solution. As a solution strategy, we perform decomposition of the optimization problem into the power control problem and scheduling problem.
- For the power control problem, we propose an optimal polynomial time algorithm based on the evaluation of the Perron–Frobenius conditions.
- For the scheduling problem, we propose a heuristic algorithm based on the maximization of the number of concurrently transmitting users within a transmission slot by maximizing the allowable interference on each user without violating their signal-to-noise-ratio (SNR) requirements.

### 1.3. Organization of the Paper

The rest of the paper is organized as follows. Section 2 describes the system model and assumptions. Section 3 presents the mathematical formulation of the minimum length scheduling problem. Section 4 provides the problem formulation and solution for the power control problem. The scheduling algorithm is given in Section 5. Section 6 provides the performance evaluation of the proposed scheduling scheme. Finally, our concluding remarks are given in Section 7.

## 2. System Model and Assumptions

**Figure 1:**
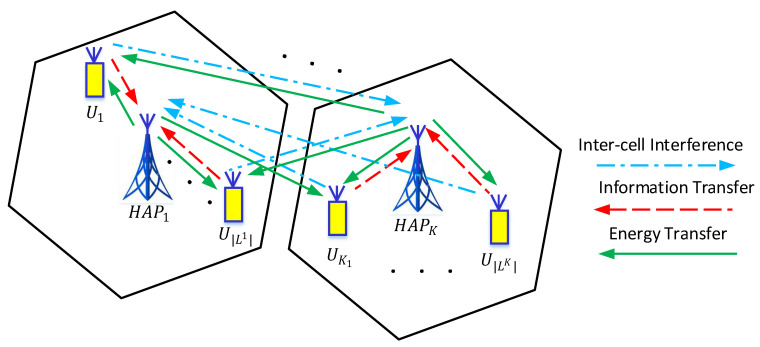
Multi-cell wireless powered communication network architecture.

The system model and related assumptions are described as follows:

- We consider a multi-cell WPCN, which consists of *K* HAPs and *N* users denoted by N as shown in Figure 1. All the HAPs and users are equipped with a full-duplex antenna. The HAPs are connected to a stable power line and transmit at a constant power $P_{h}$ continuously. On the other hand, users do not have any external power supply and can only harvest energy from the HAPs. The harvested energy is stored in a rechargeable battery with initial level $B_{n}$ for user *n*, where n∈N, and capacity $B_{max}$. The users can harvest energy from all the HAPs but only transmit information to a single HAP to which they are connected to. The set of users connected to HAP *k* is denoted by $L^{k}$ for k∈{1,⋯,K} such that ${⋃}_{k=1}^{K}L^{k}=N$. The important symbols used in this paper and their definitions are given in Table 1.
- We consider the time division multiple access (TDMA) protocol within each cell for the uplink information transmission. The TDMA is more power efficient since it allows the users to keep their transmitter circuitry inactive until their particular allocated time slot. One of the HAP works as the central HAP, i.e., central gateway and network manager in WirelessHART or system manager in International Society of Automation (ISA) 100.11a [31]. During the initialization phase, all the HAPs collect the channel state information (CSI) from the users and share it with the central HAP. The central HAP then runs the algorithm and shares the resulting resource allocation, scheduling decisions, and synchronization information by using a beacon transmitted to all the HAPs.
  Since the focus of this study is to design a resource allocation and scheduling algorithm, the mechanisms for synchronization are out of scope of this paper and can be found in [32]. The total time in which the system remains operational is partitioned into frames, and each frame is further divided into *M* variable length non-overlapping time slots. In each time slot, a subset of users from different cells transmits their information simultaneously to their respective HAP. No intra-cell interference exists except the self interference at the HAP due to full duplex operational mode. However, simultaneous transmitting users from different cells may create interference to each other.
- The uplink information transmission and downlink energy transfer channels are assumed to be different. The uplink channel gain from user *n* to HAP *k* is denoted by $g_{nk}$, and the downlink channel gain from HAP *k* to user *n* is denoted by $h_{nk}$. We assume that all the channels are quasi-static, i.e., the channel gains remain the same in the current frame and can vary independently in the next frame [12,33,34,35]. We further assume that the channel state information is perfectly known at the HAP [8,35,36].
- We assume that user *n* has a traffic demand $D_{n}$ bits to be transmitted in each frame.
- We assume that each user allocated to the transmission slot *m* transmits information at constant rate *r* if the SNR of user *n* is above a fixed threshold $\gamma_{n}$ as
  (1) $$\frac{P_{n}g_{nn}}{N_{o}W+W\sum_{j\neq n}P_{j}g_{jn}+\beta P_{h}}\geq \gamma_{n},\;n\in N$$
  where $P_{n}$ is the transmit power of user *n*, *W* is the channel bandwidth, $N_{o}$ is the noise density, $\beta P_{h}$ represents the interference created by the energy radiation by the HAPs including self-interference and interference from the other HAPs, and the term $W\sum_{j\neq n}P_{j}g_{jn}$ is the interference at the HAP to which user *n* is connected from other users that are concurrently transmitting information in slot *m*.
- We consider a realistic non-linear energy harvesting model [37], in which the energy harvesting rate for user *n* is
  (2) $$C_{n}=P_{s}\left[\Psi_{n}-\Omega_{n}\right]{\left[1-\Omega_{n}\right]}^{-1}$$
  where $\Omega_{n}={\left(1+e^{a_{n}b_{n}}\right)}^{-1}$ is a constant to guarantee zero-input zero-output response; $P_{s}$ is the maximum harvested power during saturation; and $\Psi_{n}={\left(1+e^{-a_{n}\left(P_{n}^{T}-b_{n}\right)}\right)}^{-1}$ is the logistic function related to user *n*. *a* and *b* are the positive constants related to the non-linear charging rate with respect to the input power and turn-on threshold, respectively. For a given energy harvesting circuit, the parameters $P_{s}$, *a* and *b* are determined by curve fitting.
- We assume that the users can harvest energy from all the HAPs, and the total power received by user *n* is given as follows:
  (3) $$P_{n}^{T}=\sum_{k=1}^{K}h_{nk}P_{h}$$

## 3. Minimum Length Scheduling Problem Formulation

The joint optimization of the power control and scheduling for concurrently transmitting users with the objective of minimizing the schedule length with the traffic demand, energy causality, and user transmit power constraints is formulated and denoted by MC−MLSP. The formulation of the constant transmission rate model is as follows:

MC−MLSP:

*minimize*

(4) $$\sum_{m=1}^{M}\tau^{m}$$

*subject to*

(5) $$\sum_{m=1}^{M}z_{n}^{m}=1,\;n\in N$$

(6) $$\sum_{n\in L^{k}}z_{n}^{m}\leq 1,\;m\in \left\{1,\cdots ,M\right\},k\in \left\{1,\cdots ,K\right\},$$

(7) $$x_{n}^{m}\tau^{m}\geq D_{n}\;n\in N,\;m\in \left\{1,\cdots ,M\right\},$$

(8) $$x_{n}^{m}=z_{n}^{m}r\;n\in N,\;m\in \left\{1,\cdots ,M\right\},$$

(9) $$\frac{P_{n}g_{nn}}{N_{o}W+W\sum_{j\neq n}P_{j}g_{jn}+\beta P_{h}}\geq \gamma_{n},\;n\in N,$$

(10) $$B_{n}+\sum_{j=1}^{m}C_{n}\tau^{j}-z_{n}^{m}P_{n}\tau^{m}\geq 0,\;n\in N,\;m\in \left\{1,\cdots ,M\right\}$$

(11) $$P_{n}\leq z_{n}^{m}P_{max},\;n\in N,\;m\in \left\{1,\cdots ,M\right\},$$

*variables*

(12) $$P_{n}\geq 0,\;\tau^{m}\geq 0,\;z_{n}^{m}\in \left\{0,1\right\},\;n\in N,\;m\in \left\{1,\cdots ,M\right\}.$$

The variables of the problem are $P_{n}$, the transmit power of user *n*; $\tau^{m}$, the length of transmission slot *m*; and $z_{n}^{m}$, a binary variable, which takes a value 1 if user *n* is allocated to slot *m* and 0 otherwise. $x_{n}^{m}$ denotes the transmission rate of user *n* in time slot *m* and *r* is the constant rate level.

The objective of the optimization problem is to minimize the schedule length as given by Equation (4). Equation (5) states that each user should be allocated to only one time slot. Equations (6) represents that, at most, one user can be allocated to a slot for the same HAP. Equations (7) and (8) together represent the traffic demand of users. Equation (9) represents the condition for SNR to achieve a constant rate. Equation (10) gives the energy causality constraint: The total amount of available energy, including both the initial energy and the energy harvested until and during the transmission of a user, should be greater than or equal to the energy consumed during its transmission. Equation (11) represents the maximum transmit power constraint for the users.

The optimization problem formulated in Equations (4)–(12) includes both continuous variables, which are $P_{n}$ and $\tau^{m}$, and integer (binary) variables, such as $z_{n}^{m}$. Furthermore, it is a non-linear problem due to the multiplication of two variables in energy causality constraint given in Equation (10). Hence, this is a Mixed Integer Non-Linear Programming (MINLP) problem, which are, in general, difficult to solve for optimal solutions in polynomial time [7].

### Solution Framework

As the optimization problem is a Mixed Integer Non-Linear Programming (MINLP) problem, finding the global optimal solution requires exponential time algorithms, which are intractable even for medium size networks [38]. To overcome this intractability, we decompose the optimization problem into the optimal power control problem and scheduling problem as detailed below:

- For a given set of concurrently transmitting users, we formulate the optimization problem to determine the minimum transmission slot length and the corresponding transmit power vector while considering the maximum transmit power constraint, traffic demand, and energy causality of the users. We first show that the power control problem is a feasibility problem, and then, by using the Perron–Frobenius condition, we find the optimal power vector if the problem is feasible.
- Determining the power vector and transmission slot length for a given set of users reduces the problem to the optimization of the concurrently transmitting users within a transmission slot. For the scheduling problem, we exploit the affordable interference levels of all the users and group them based on these interference levels while considering the maximum transmit power and energy causality of the users.

## 4. Power Control Problem

In this section, we determine the optimal power control and time slot length for a given set of concurrently transmitting users from different cells with the objective of minimizing the time slot length. For simplification, we remove the superscript *m* in Section 3, which represents the time slot index. The optimal power control problem is formulated as follows: *minimize*

(13) t

*subject to*

(14) $$\frac{D_{n}}{x_{n}}\leq t,\;n\in S,$$

(15) $$B_{n}+C_{n}t-P_{n}t\geq 0,\;n\in S,$$

(16) $$P_{n}\leq P_{max},\;n\in S,$$

(17) $$x_{n}=r,\;n\in S,$$

(18) $$\frac{P_{n}g_{nn}}{N_{o}W+W\sum_{j\neq n}P_{j}g_{jn}+\beta P_{h}}\geq \gamma_{n},\;n\in S$$

*variables*

(19) $$P_{n}\geq 0,\;t>0,n\in S.$$

The objective of this problem is to determine the optimal time slot length for a given set of concurrently transmitting users in S. The constraints in Equations (14)–(18) represent the traffic demand, energy causality, maximum transmit power, and constant transmission rate constraint, respectively. Note that $B_{n}$ refers to the energy available at the start of the transmission slot, and we used the same notation for simplicity.

This optimization problem can be reduced to a feasibility problem. If a feasible power vector exists, then the optimal time slot length is $t=\max_{n\in S}D_{n}/r$. In the following, we present the feasibility of the concurrent transmission of the users in set S. For the feasibility of concurrent transmission, there should exist a set of transmit power levels $P_{n},\forall n\in S$, such that the SNR constraint given in Equation (18) is satisfied for each user in S without violating the maximum transmit power and energy causality constraints given in Equations (15) and (16).

At any decision time $t_{dec}$, the existence of such a set of transmit power levels that satisfy the $P_{max}$ constraint can be determined by using Perron–Frobenius conditions described as follows: Let $G_{S}$ be a |S|×|S| relative channel gain matrix, in which $a_{ij}=g_{ji}/g_{ii}$ represents the entry of the *i*th row and *j*th column for i≠j and $a_{ij}=0$ for i=j. Let $D_{S}$ represent a |S|×|S| diagonal matrix with the *i*th entry equal to $\gamma_{i}$.

Let $\sigma_{S}$ be a |S|×1 normalized noise power vector with the *i*th entry equal to $\gamma_{i}WN_{0}/g_{ii}$. Then, the Perron–Frobenius condition states that there exists a power allocation vector that satisfies the $P_{max}$ constraint if and only if the largest real eigenvalue of $D_{S}G_{S}$ is less than 1 and every element of the component-wise minimum power vector ${\left(I-D_{S}G_{S}\right)}^{-1}\sigma_{S}$ is less than or equal to $P_{max}$, i.e., ${\left(I-D_{S}G_{S}\right)}^{-1}\sigma_{S}⪯P_{\max}$, where $P_{\max}$ is a vector with all entries equal to $P_{max}$.

For a feasible solution, the users must also satisfy the energy causality constraint, therefore, if the transmit powers also follow the energy causality constraint, the solution is feasible at the time of the decision.

**Lemma** **1.** *If $P^{\min}={\left(I-D_{S}G_{S}\right)}^{-1}\sigma_{S}$ is an infeasible solution to problem in Equations (13)–(19), then, there is no feasible solution.*

**Proof.**  Let $P^{\min}=\left\{P_{1}^{min},\cdots ,P_{\left|S\right|}^{min}\right\}$ denote the minimum power vector for a set S. Any other power vector $P^{\prime }$ satisfying the SNR constraint is component-wise greater than $P^{\min}$, i.e., $P^{\prime }≻P^{\min}$. Then, if $P^{\min}$ is infeasible, there exists either $P_{k}^{min}>P_{max}$ for any particular user k∈S or required energy $E_{j}$ is greater than the available energy, i.e., $E_{j}=P_{j}^{min}D_{j}/r>E_{j}^{available}$ for any particular use *j*. Then, if $P^{\min}$ is infeasible, there is no feasible solution for problem in Equations (13)–(19), since either $P_{k}^{{}^{\prime }}$ will be greater than $P_{max}$ or $E_{j}^{{}^{\prime }}$ will be still greater than $E_{j}^{available}$, making $P^{\prime }$ also infeasible.  □

The following theorem states that a feasible $P^{\min}$ vector is an optimal solution to the optimization problem in Equations (13)–(19).

**Theorem** **1.** *Let $P^{\min}={\left(I-D_{S}G_{S}\right)}^{-1}\sigma_{S}$ denote the minimum feasible power vector obtained from Perron–Frobenius conditions. Then, if $P^{\min}$ is feasible, i.e., $P^{\min}⪯P_{\max}$, and it satisfies the energy causality constraint of each user, then, $P^{\min}$ is an optimal solution to optimization problem in Equations (13)–(19).*

**Proof.**  Any feasible power vector gives the same solution to problem in Equations (13)–(19), i.e., $t=\max_{n\in S}D_{n}/r$. Thus, if $P^{\min}$ is feasible, it is an optimal solution.  □

Since the computational complexity of evaluating Perron–Frobenius conditions for |S| users is $O(|S{|}^{3})$, the computational complexity of solving the power control problem for the constant transmission rate model is $O(|S{|}^{3})$.

## 5. Scheduling

In Section 4, we solved the power control problem optimally for a known set of users transmitting information simultaneously. For all the users transmitting concurrently, the information transmission may be too long due to the extensive interference caused to each other. Therefore, the schedule length can further decrease by intelligently grouping the users into a set of subsets for concurrent transmission. However, determining the optimal subsets of the users transmitting concurrently and their schedule requires an exponential computational effort. Let $\nu =\{\nu_{j}:1\leq j\leq |\nu |\}$ denote the set of all possible partitions of the set of users N={1,⋯,N}, where a partition of set N is defined as a set of nonempty mutually exhaustive and mutually exclusive subsets of set N, e.g., for the set of 10 users, {{1,5,8},{4,7,9,10},{2,3,6}} is one possible partition of set N.

The total number of such partitions for a set of *N* users is |ν|=∑j=0N−1N−1jBj, where $B_{j}$ is a Bell number with $B_{0}=1$ and Bj=∑z=0j−1j−1zBz. For a given partition of N, each subset of users transmits information concurrently within a time slot. Therefore, for a specific partition $\nu_{j}$ with $\zeta_{j}$ number of subsets, the total number of possible transmission sequences are $\zeta_{j}!$, i.e., for the above mentioned example $\zeta_{j}=3$ resulting in six possible transmission sequences.

Then, total number of possible transmission sequences for a set of users N is $\sum_{j=1}^{\left|\nu \right|}\zeta_{j}!$. This exponential computational complexity of the scheduling problem makes the problem intractable even for medium size networks. Hence, scalable and fast solutions are required. In the following, we present the scheduling problems.

In this section, we present a heuristic scheduling algorithm, which aims at maximizing the number of users within each concurrently transmitting set by maximizing the interference on each user without violating the energy causality, SNR requirements, and maximum transmit power constraint.

Let $I_{n}^{max}$ denote the maximum allowed interference level from other users that a particular user *n* can afford, i.e., any interference level greater than $I_{n}^{max}$ results in an infeasible transmission scenario for user *n*. This maximum allowed interference can be determined by using Equation (9) as follows:

(20) $$I_{n}^{max}=\frac{1}{W}\left[\frac{P_{n}g_{nn}}{\gamma_{n}}-N_{0}W-\beta P_{h}\right],$$

It is important to note that the maximum allowed interference level of any user is an increasing function of the transmit power of that user. Hence, increasing the transmit power allows the user to accommodate more interference and possibly the allocation of more simultaneously transmitting users in a slot.

Furthermore, for a user *n*, the earliest scheduling time $ts_{n}$ is defined as the first time instant when it can afford transmission at constant rate individually and is given by $ts_{n}=\max\left(0,P_{n}^{min}D_{n}/C_{n}r-B_{n}/C_{n}-D_{n}/r\right)$, where $P_{n}^{min}=\gamma_{n}\left(N_{0}W+\beta P_{h}\right)/g_{nn}$, is the minimum power required to achieve the SNR $\gamma_{n}$ without any interference, which is derived from Equation (18) for the case when the user transmits individually. In the following analysis, we assume that $P_{n}^{min}\leq P_{max}$ exists, otherwise, the problem is infeasible. The addition of any user in set S results in a delay in the earliest time instant $ts_{n}$ due to increased interference.

The scheduling algorithm should determine the subsets of users such that the maximum number of users transmit their information simultaneously with the interference level close to the $I_{n}^{max}$ for each user *n*. Therefore, at any decision time $t_{dec}$, first, we determine a set of users that can afford the constant rate at $t_{dec}$, i.e., the users for which $ts_{n}\leq t_{dec}$. Then, for this set of users, we evaluate the maximum power that they can afford, i.e., $P_{n}=max\left(P_{max},E_{n}\left(t_{dec}\right)/\left(D_{n}/r\right)\right)$ and the maximum level of interference they can afford from other users in a concurrent transmission, i.e., $I_{n}^{max}=\frac{1}{W}\left(P_{n}g_{nn}/\gamma_{n}-N_{0}W-\beta P_{h}\right)$.

Then, CRSA evaluates the pairwise interference of the feasible users and groups the users from different cells such that the number of concurrently transmitting users within each time slot is maximized without violating the maximum allowed interference level.

The Constant Rate Scheduling Algorithm (CRSA) is given in Algorithm 1 and described in detail next. Let $S=\{S^{1},\cdots ,S^{M}\}$ be a set of subsets of users concurrently transmitting in time slots $t^{*}=\left\{t_{1}^{*},\cdots ,t_{M}^{*}\right\}$ by using powers $P^{*}=\left\{P_{1}^{*},\cdots ,P_{M}\right\}$. The *m*th entry of S denoted by $S^{m}$ contains a subset of users that transmit simultaneously in the *m*th time slot with duration $t_{m}^{*}=\max_{s\in S^{m}}D_{s}/r$ and corresponding power vector $P_{m}^{*}$.
**Algorithm 1** Constant Rate Scheduling Algorithm (CRSA)**Input**: A set of users N**Output**: Simultaneously transmitting user set in each slot S, transmission time for eachslot $t^{*}$, transmit power vector $P^{*}$ 1: 
m←1, $t_{dec}\leftarrow 0$, P←0, $I^{\max}\leftarrow 0$, I←0 2: 
determine $ts_{n}$, for all n∈N
 3: 
**while** 
N≠∅
 4:     
$F\leftarrow \emptyset ,S^{m}\leftarrow \emptyset$
 5:     
**if** 
$t_{dec}<\min_{n\in N}ts_{n}$
 6:         
$t_{dec}=\min_{n\in N}ts_{n}$
 7:     
**end if** 
 8:     
**for** 
n∈N
 9:         
**if** 
$ts_{n}\leq t_{dec}$
10:            
F←F+n
11:            
$E_{n}\left(t_{dec}\right)=B_{n}+C_{n}\left(t_{dec}+D_{n}/r\right)$
12:            
$P_{n}\leftarrow min\left(P_{max},E_{n}\left(t_{dec}\right)/\left(D_{n}/r\right)\right)$
13:            
$I_{n}^{max}\leftarrow \frac{1}{W}\left[\frac{P_{n}g_{nn}}{\gamma_{n}}-N_{0}W-\beta P_{h}\right]$2: 
14:         **end if**15:     **end for**16:    
evaluate interference $I_{ns}$ of user *n* to *s*, for all n,s∈F17:    
$u\leftarrow arg\max_{j\in F}I_{j}^{max}$
18:    
 $I_{s}\leftarrow 0$, for all s∈F
19:    
 $S^{m}\leftarrow S^{m}+\left\{u\right\}$
20:     
**for** k=1:K
21:         
**if** u∉$L_{k}$
22:            
 $F^{k}\leftarrow L_{k}\cap F$
23:             
sort $F^{k}$ in descending order of $I_{n}^{max}$
24:             
**for** $v\in F^{k}$ 
25:                 
**if** 
$\sum_{j\in S^{m}}I_{jv}\leq I_{v}^{max}$
26:                       
**if** 
$I_{s}^{max}-I_{s}\geq I_{vs},\;\forall s\in S^{m}$
27:                           
$I_{v}\leftarrow \sum_{j\in S^{m}}I_{jv}$
28:                           
$I_{s}=I_{s}+I_{vs},\;\forall s\in S^{m}$
29:                           
$S^{m}\leftarrow S^{m}+F^{k}\left(v\right)$
30:                           
break,
31:                       **end if**
32:                   **else**
33:                     
break,
34:                   **end if**
35:               **end for**
36:           **end if**
37:     **end for**
38:     
$t_{m}^{*}\leftarrow \max_{s\in S^{m}}D_{s}/r,$
39:     $N\leftarrow N-S^{m}$
40:     $t_{dec}=t_{dec}+t_{m}^{*}$
41:     m←m+1
42:  **end while**


Let $I^{\max}$ be a N×1 maximum affordable interference vector, in which *j*-th entry represents the maximum allowed interference level from other users on user *j*. Let I be a N×K pairwise interference matrix, in which $I_{ij}$ is the entry of *i*th row and *j*th column representing the interference caused by user *i* on the HAP *j*.

The input of CRSA is a set of users denoted by N, whereas its outputs include a set S, and corresponding time and power allocation vectors denoted by $t^{*}$ and $P^{*}$, respectively. The algorithm starts by initializing the transmission slot number *m* to 1, decision time $t_{dec}$ to 0, power vector P, maximum affordable interference $I^{\max}$, and interference matrix I to 0 (Line 1). Then, it determines the earliest starting time $ts_{n}$ for every user n,n∈N (Line 2).

In each iteration, the algorithm determines the set $S^{m}$ as well as their powers. The CRSA first updates the decision time by setting it equal to the minimum of earliest starting times of the users, since no user has enough energy for transmission earlier than (Lines 5–7). Then, it checks the feasibility of each unallocated user based on their earliest scheduling time (Line 9). For the set of feasible users at time $t_{dec}$, denoted by F, CRSA evaluates the amount of energy each user needs to complete its transmission, the maximum transmit power, and the maximum interference each user can afford (Lines 11–13).

Once the set of feasible users F and their parameters are determined, the algorithm groups the users in set F with similar interference characteristics for concurrent transmission. For this purpose, the algorithm evaluates the pairwise interference that each user in F causes to others while transmitting simultaneously (Line 16). CRSA picks the user that can afford the maximum interference from other users, since this allows the addition of more users in the set of simultaneously transmitting users (Line 17).

Then, it initializes the interference that is created on a certain user by concurrent transmissions $I_{s}$ to 0 and adds the user that can afford maximum interference to the set $S^{m}$ for transmission in the current time slot (Lines 18–19). The algorithm attempts to group the most suitable user from each cell iteratively (Lines 20–37). For this, CRSA visits each cell one by one and terminates the search when either a feasible user is found or no user can be grouped from the current cell.

For each cell, CRSA first sorts all the feasible users within the cell based on their $I_{n}^{max}$ values (Line 23). Then, it checks the compatibility for each user for possible concurrent transmission until it finds a suitable user from current cell. For a user *v* to be compatible for concurrent transmission in the set $S^{m}$, the total interference caused by all the users in set $S^{m}$ must be less than $I_{v}^{max}$, i.e., $\sum_{j\in S^{m}}I_{jv}\leq I_{v}^{max}$ (Line 25), and the interference caused by user *v* must be less than the affordable interference margin of the existing users in set $S^{m}$, i.e., $I_{s}^{max}-I_{s}\geq I_{vs},\;\forall s\in S^{m}$ (Line 26).

If the first condition is not valid for the user with maximum $I_{n}^{max}$ value in the current cell, this implies that no other user from current cell can afford this much interference, and hence the algorithm moves to the next cell without picking any user from this cell (Line 33). If the first condition is true but the user is causing excessive interference to the users in set $S^{m}$, the algorithm continues searching among the remaining feasible users within current cell for simultaneous transmission (Lines 24–32).

If both of these conditions are true, the algorithm updates the interference levels of set $S^{m}$, adds this user to set $S^{m}$ and moves to the next cell (Lines 27–29). Once all the cells are visited, CRSA evaluates the transmission time slot length for $S^{m}$, updates the set of unallocated users, the decision time, and the slot number (Lines 38–41). The algorithm terminates when all the users are allocated (Line 3).

Note that in half-duplex, the transmission frame is divided into two non-overlapping phases named the energy harvesting phase, in which all users harvest energy, and the information transmission phase, in which the users transmit their information by using the harvested energy. Due to fixed and equal energy harvesting duration for all users, the scheduling is not that important, i.e., the order of transmissions in the information transmission phase in not relevant since the users are not harvesting energy during the information transmission time of other users.

On the other hand, in full-duplex, since the users can harvest energy during the transmission of previously allocated users, this allows us to reduce the schedule length by intelligently scheduling the users. If we eliminate the impact of self-interference, the proposed algorithm can be used for a half duplex system with minor modifications as follows. The users can be grouped by exploiting the maximum affordable interference from other users as performed in CRSA algorithm, and the energy harvesting duration $\tau_{0}^{m}$ can be determined for each group by using the half-duplex assumption in which no energy is harvested during the information transmission phase. Then, the overall energy harvesting duration will be $\max\tau_{0}^{m}$ for m∈{1,2,⋯,M}.

## 6. Simulation Results

The goal of this section is to evaluate the performance of the proposed scheduling algorithm in comparison to the multi-cell transmission with no scheduling and a previously proposed penalty-based scheduling algorithm for a single cell network. The multi-cell transmission with no scheduling, denoted by MCNS, randomly selects a single user from each cell for the concurrent transmission by using the constant rate within a transmission slot. The previously proposed penalty-based scheduling algorithm in [24], denoted by MPA, aims to minimize the schedule length for a single cell full-duplex WPCN by scheduling the users in increasing order of their penalties.

The penalty function of user *i* is defined as the difference between the actual transmission time and the minimum possible transmission time at any decision time, where the minimum possible transmission time of user *i* is the time corresponding to maximum transmit power, i.e., $P_{i}=P_{max}$. MPA uses the continuous rate model, in which the Shannon channel capacity formula for an additive white Gaussian noise (AWGN) channel is used to calculate the transmission rate of the users as a function of the SNR. For our simulations, MPA picks the user with the minimum penalty among all the users within all cells and allocates it to the current time slot without allowing concurrent transmissions.

Simulation results are obtained by averaging 1000 independent random network realizations. The cells are uniformly distributed within a circle with radius 100 m, and the users are uniformly distributed within the coverage of each cell with radius 10 m. The attenuation of the links considering large-scale statistics are determined by using the path loss model given by

(21) $$PL\left(d\right)=PL\left(d_{0}\right)+10\alpha log_{10}\left(\frac{d}{d_{0}}\right)+Z,$$

where PL(d) is the path loss at distance *d*, $d_{0}$ is the reference distance, α is the path loss exponent, and *Z* is a zero-mean Gaussian random variable with standard deviation σ. The small-scale fading is modeled using Rayleigh fading with the scale parameter Ω set to the mean power level obtained from the large-scale path loss model. The parameters used in the simulations are $D_{n}=100$ bits for n∈N and the constant rate r=50 Kbps. The constant rate and corresponding SNR are linked to each other by the Shannon capacity formulation; W=1 MHz; $d_{0}=1$ m; $PL(d_{0})=30$ dB; α=2.7, σ=4 [8,24]. The self interference coefficient β is −70 dBm. The initial battery levels of the users are $10^{-9}$ J, $P_{max}=1$ mW and $P_{h}=1$ W for the simulations, unless otherwise stated [24].

**Figure 2:**
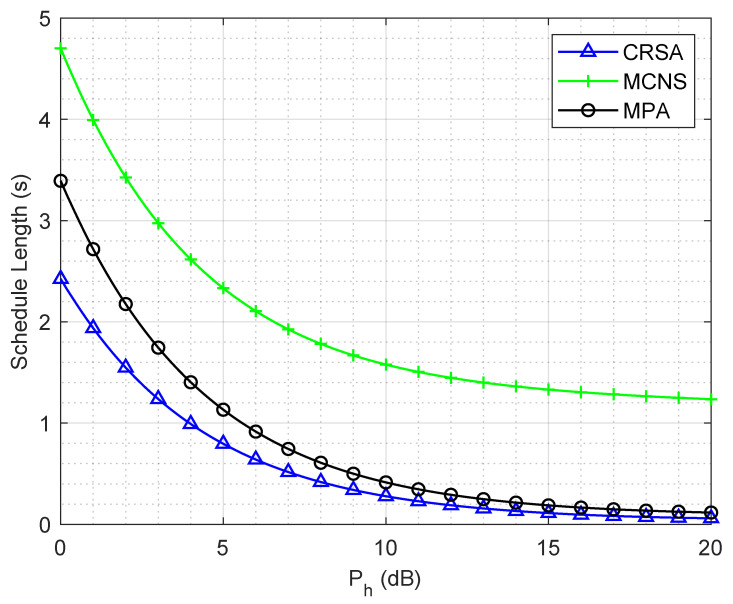
Schedule length of the algorithms for different HAP transmit powers in a network of 10 cells and 50 users.

Figure 2 shows the schedule length of the algorithms for different HAP transmit powers in a network of 10 cells and 50 users. The schedule length decreases as the HAP transmit power increases, since higher HAP power allows the users to harvest more energy, which allows users to reach the constant rate earlier and, hence, a lower waiting time. For lower values of HAP power, the schedule length of CRSA is 50% less than MCNS, since CRSA considers the concurrent transmission of users while considering their energy harvesting rates and interference levels.

On the other hand, MCNS randomly selects users for concurrent transmission without considering the energy harvesting rates and interference characteristics, which may lead to grouping the users with very low energy harvesting rates or severe interference levels, resulting in longer slot lengths. The MPA has a higher schedule length than CRSA, although the MPA uses the continuous rate model, which has a natural advantage over the constant transmission rate model; however, CRSA still performs better. Since CRSA considers the concurrent transmissions while MPA only schedules a single user in each time slot, i.e., no concurrent transmissions. It is important to note that CRSA gives a lower schedule length in comparison to MPA due to the proper scheduling of the users for concurrent transmission.

**Figure 3:**
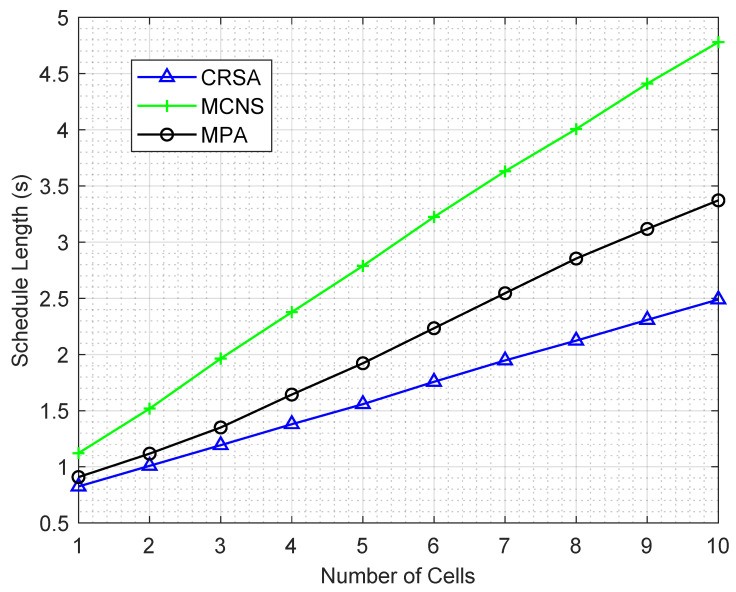
Schedule length of the algorithms for different number of HAPs with five users in each HAP.

Figure 3 shows the performance of the algorithms for different number of cells in a network with five users in each cell. The schedule length increases as the number of cells increases in the network, since a higher number of cells results in a higher traffic load, which requires more transmission combinations due to the different interference and energy harvesting rates of the users. For a single cell network, MPA and CRSA almost perform similar. This is due to the fact that there are no concurrent transmissions in a single cell network.

The schedule length of CRSA is half of MCNS and 30% lower than MPA for larger networks due to the intelligent grouping of the users for concurrent transmission. For the MCNS, the steep increase in the schedule length is due to the fact that MCNS randomly selects one user from each cell for simultaneous transmission, which may cause severe interference to other users. For MCNS and MPA algorithms, the addition of a new cell results in an almost constant increase. This is due to the fact that the addition of a new cell increases the number of users in the network and, hence, interference for the concurrent transmission.

For CRSA, the addition of a new cell results in less increase in the schedule length compared to other algorithms. This is because the CRSA attempts to allocate more users within a transmission slot; therefore, the user from a new cell is mostly accommodated within the same transmission slot without any increase in the transmission slot length.

**Figure 4:**
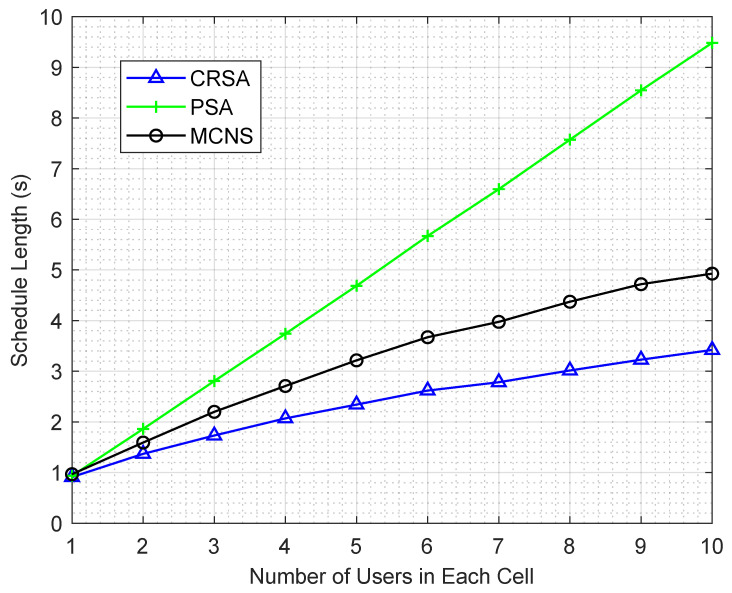
Schedule length of the algorithms for different number of users in each cell in a network of 10 HAPs.

Figure 4 illustrates the impact of network size on the schedule length for the algorithms. The schedule length increases as the number of users increases within each cell, since this increase requires more transmission slots to complete the transmission of all the users. The schedule length of MCNS increases almost linearly as the number of users increases in a cell. On the other hand, the effect of each new user on the CRSA algorithm diminishes, since a higher number of users within each cell increases the probability of finding a more suitable user for the concurrent transmission, resulting in smaller schedule length.

**Figure 5:**
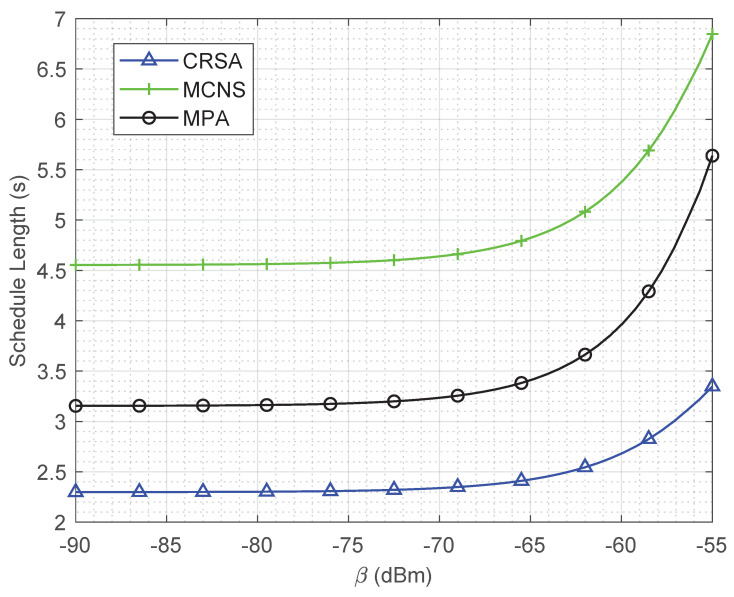
Schedule length of the algorithms for different interference levels in a network of 10 cells and 50 users.

Figure 5 illustrates the schedule length for different values of self-interference coefficient β. For lower values of β, i.e., −90 to −70 dBm, the schedule length is constant due to the low impact of self-interference. Since the self-interference is very small as compared to noise value; therefore, the SNR values do not change too much to affect the required energy and transmission time. However, as the β increases, the schedule length increases drastically due to the dominating effect of self-interference as compared to the noise power. Due to this reason, the SNR drops quickly resulting in a lower probability of concurrent transmissions. It is important to note that, due to intelligent scheduling of the users, the schedule length of CRSA is almost half of the MCNS for higher values of β.

## 7. Conclusions

In this paper, we formulated a mixed integer programming problem for the minimum length scheduling problem to determine the power control and scheduling, which is difficult to solve for the global optimal solution. As a solution strategy, we decomposed the optimization problem into the power control and scheduling problems. First, we solved the power control problem based on the evaluation of Perron–Frobenius conditions. Then, the proposed optimal power control solution was used to determine the optimal transmission time for a subset of users that will be scheduled by the scheduling algorithm.

For the scheduling problem, we proposed a heuristic algorithm based on the maximization of the number of concurrently transmitting users by maximizing the allowed interference on each user. Through extensive simulations, we demonstrated that the proposed solutions outperformed the conventional successive transmission and concurrent transmission of randomly selected users for different HAP transmit powers, network densities, and network size.

## Figures and Tables

**Table 1:** Symbols and notations.

Symbol	Definition
*N*	Total number of users
*K*	Total number of HAPs
*M*	Total number of time slots
$g_{nk}$	Uplink channel gain from user *n* to HAP *k*
$h_{nk}$	Downlink channel gain from HAP *k* to user *n*
$\tau^{m}$	The length of transmission slot *m*
$x_{n}^{m}$	Transmission rate of user *n* in slot *m*
$z_{n}^{m}$	Time slot allocation variable
$D_{n}$	Data requirement of user *n*
$r^{i}$	Transmission rate of user *i*
$\gamma_{n}$	Signal-to-noise ratio threshold for user *n*
$P_{h}$	Transmit power of HAP
$\beta P_{h}$	Power of Self-interference at HAP
$P_{n}$	Transmit power of user *n*
$I_{ij}$	Interference of user *i* on HAP *j*
$P_{max}$	Maximum transmit power of a user
$C_{n}$	Energy harvesting rate of user *n*
$B_{n}$	Initial battery level of user *n*
$I_{n}^{max}$	Maximum allowed interference level for user *n*
S	Set of concurrently transmitting users
*W*	Bandwidth
$N_{0}$	Noise density
$P_{n}^{T}$	Total received power by user *n*
$E_{n}$	Energy of user *n*
α	Path loss exponent

## Data Availability

Not applicable.
